# The Controversy about a Possible Relationship between Mobile Phone Use and Cancer

**DOI:** 10.1289/ehp.11902

**Published:** 2008-09-26

**Authors:** Michael Kundi

**Affiliations:** Institute of Environmental Health, Medical University of Vienna, Vienna, Austria

**Keywords:** acoustic neuroma, brain tumors, causation, epidemiology, glioma, meningioma, mobile phones, risk assessment

## Abstract

**Objective:**

During the last decade, mobile phone use increased to almost 100% prevalence in many countries of the world. Evidence for potential health hazards accumulated in parallel by epidemiologic investigations has raised controversies about the appropriate interpretation and the degree of bias and confounding responsible for reduced or increased risk estimates.

**Data Sources:**

Overall, I identified 33 epidemiologic studies in the peer-reviewed literature, most of which (25) were about brain tumors. Two groups have collected data for ≥ 10 years of mobile phone use: Hardell and colleagues from Sweden and the Interphone group, an international consortium from 13 countries coordinated by the International Agency for Research on Cancer.

**Data Synthesis:**

Combined odds ratios (95% confidence intervals) from these studies for glioma, acoustic neuroma, and meningioma were 1.5 (1.2–1.8); 1.3 (0.95–1.9); and 1.1 (0.8–1.4), respectively.

**Conclusions:**

Methodologic considerations revealed that three important conditions for epidemiologic studies to detect an increased risk are not met: *a* ) no evidence-based exposure metric is available; *b*) the observed duration of mobile phone use is generally still too low; *c*) no evidence-based selection of end points among the grossly different types of neoplasias is possible because of lack of etiologic hypotheses. Concerning risk estimates, selection bias, misclassification bias, and effects of the disease on mobile phone use could have reduced estimates, and recall bias may have led to spuriously increased risks. The overall evidence speaks in favor of an increased risk, but its magnitude cannot be assessed at present because of insufficient information on long-term use.

Because of the enormous increase in mobile phone use starting in the mid-1990s and reaching almost 100% prevalence in many countries worldwide by now, concerns have been raised that even small risks for developing chronic diseases such as cancer from mobile phone use may have substantial impact on public health. In fact, never before in history has any device of comparative prevalent use been associated with such high exposure to high-frequency electromagnetic fields (EMFs). (In addition, exposure to extremely low-frequency magnetic fields occurs from battery discharge.) Although from the perspective of the thermal effects paradigm, the rate of energy deposition in tissues of the mobile phone user is below levels considered harmful, there has been debate since the 1930s that tissue heating may not be the only relevant effect elicited by exposure to high-frequency EMFs; thus, there may be a relevant risk that has not been established yet because of the scarcity of exposure conditions that are comparable across a significant proportion of the population. For all the diverse high-frequency exposures occurring in environ mental and occupational settings ranging from long-waves [a type of amplitude modulation (AM) broadcasting with carrier frequencies between 153 and 280 kHz] to radar waves, only a few long-term observational studies have been published (for an overview, see Ahlbom et al. 2004; Krewski et al. 2001; Kundi et al. 2004). Similarly scarce are long-term animal studies of low-level exposures in the pre–mobile phone era. Hence, at the time mobile phones were introduced, there were insufficient data to decide about health risks from low-level exposures, but the prevailing opinion that no rele vant health effects occur at exposures below guideline levels led to the expectation that mobile phones are safe. However, the exponential growth of mobile phone use came as a surprise to the industry as well as to scientists involved in EMF risk assessment. Therefore, the existing gaps in knowledge should be addressed in both experimental as well as epidemiologic investigations focusing on exposures occurring in mobile telecommunications.

Since the mid-1990s, many epidemiologic studies of mobile phone use have been conducted worldwide, most of them focusing on tumors of the head region. Despite the growing database, the concerns have not been settled and a controversy still exists about possible adverse health effects. Although some may be inclined to attribute the ongoing debate to the enormous economic impact of modern telecommunication during the past decade, this debate also mirrors fundamental difficulties and a permissible range of interpretation under circumstances of insufficient knowledge. Despite this state of affairs, not all arguments that have been put forward in this controversy are valid. In the following sections I will concentrate on epidemiologic findings and their interpretation, although experimental work deserves similar critical appraisal.

I will first give a brief overview of the results of epidemiologic investigations of mobile phone use and tumors of the head region. I will then address methodologic problems associated with these studies and, by application of the pragmatic approach proposed earlier (Kundi 2006), discuss whether epidemiologic evidence supports a causal interpretation of an association between mobile phone use and brain tumors.

## Overview of Epidemiologic Studies

Table 1 presents an overview of results of epidemiologic studies on the association between brain tumors and mobile phone use. Other end points include salivary gland tumors (Hardell et al. 2004; Lönn et al. 2006; Sadetzki et al. 2008), uveal melanoma (Stang et al. 2001), non-Hodgkin lymphoma (Hardell et al. 2005c; Linet et al. 2006), facial nerve tumors (Warren et al. 2003), and testicular cancer (Hardell et al. 2007), but for these diseases the database is insufficient to date.

Except for the early cohort study from the United States (Dreyer et al. 1999) that was stopped by the court after 1 year of follow-up and the Danish retrospective cohort study (Johansen et al. 2001; Schüz et al. 2006b), all investigations have been case–control studies.

The “Overall results” from 25 epidemiologic studies listed in Table 1 do not demonstrate support for an increased risk. Only a few risk estimates are significantly elevated, and some are even significantly reduced. Risk estimates for longer duration of use are higher, on average, than overall estimates, and estimates for ipsilateral mobile phone use (i.e., use of the mobile phone on the same side where the tumor occurred), where available, tend to be even higher. Implications of these findings are discussed below.

## Methodologic Problems

Although a number of established study designs in epidemiology have been successfully applied in thousands of investigations in the past 50 years, perhaps few epidemiologists are fully aware of the conditions necessary to detect an existing risk by application of these methodologies. Analytical epidemiology intends to estimate the risk as a function of exposure to an agent by application of one of three classical study types: cross-sectional, case–control, and cohort study designs. Theoretically, all three types are capable of detecting an existing risk under ideal conditions but differ in their sensitivity to the effects of extraneous and confounding factors.

For all study designs, it has to be assumed that exposure to the agent can be assessed with a certain sensitivity and specificity. In the case of mobile phone use, the appropriate exposure metric is unknown. Absorption of electromagnetic energy in the body of the user depends on technical features of the phone and the network, as well as on anatomical features and habits of use. At first glance, this situation does not seem to be much different from, for example, an exposure to an air pollutant that also varies in time and space and which internal dose will depend on physiologic conditions. The problem with mobile telephones is much more profound. What aspect of the complex exposure condition described by “mobile phone use” could be responsible for an effect? It is obvious that given a certain indicator of mobile phone use, such as years of regular use, cumulative number of calls, cumulative hours of use, or cumulative absorption of electromagnetic energy in a certain area of the body, there are indefinitely many exposure conditions that are equivalent under the chosen metric and hence induce an equivalence relation in the space of exposure patterns that cannot be assessed as to its suitability with respect to any outcome measure without a sound mechanistic theory.

Another essential problem is related to the long induction periods and latencies of tumors in the head and neck region. Mobile phone use that was insignificant before the mid-1990s could not be studied with respect to its influence during induction period because, in almost all users, malignant transformation has likely occurred long before exposure to mobile phones commenced. Although an influence during the initiation phase cannot be ruled out, many researchers have expressed the opinion that if there is an effect at all, it is an effect on tumor promotion or progression (Johansen et al. 2001; Muscat et al. 2000; Stang et al. 2001). If this is actually the case, several scenarios have to be considered. For example, in a summary of acoustic neuroma, Mohyuddin et al. (2003) found that tumor growth is exhibited in 48–70% of patients, stable tumor volumes in 27–50%, and involution in 2–10%. If mobile phone use influences growth rate, this influence could result in either restarting growth in stable tumors, an increase of growth rate in growing tumors, or an inhibition of involution. The net result would be an earlier onset of symptoms and an earlier diagnosis. If there is an effect of the specified type, the age incidence function in the exposed population would be shifted compared with that of the unexposed population by a fraction of the exposure duration, as long as duration of use is short compared with the natural history of the disease. Given that the age incidence function has a positive slope, this shift is equivalent to an increased incidence in the exposed population for any given age. For simplicity, assume that the duration and effect of exposure is constant across all age groups. Taking the slope for the age incidence function for brain tumors as 0.04 [on the log incidence scale (Wrensch et al. 2002)], the estimated incidence of brain cancer at any given age would be proportional to exp(age × 0.04) in the population as a whole. If the exposed segment of the population has an age incidence function shifted by 2 years, the estimated incidence at any given age among the exposed would be proportional to exp[(age + 2) × 0.04], resulting in an OR of exp(2 × 0.04) = 1.08 for mobile phone use. Note that the result is independent of the exposure prevalence and depends only on the shift of the age incidence function and its slope. Given the lower number of mobile phone users at an older age, the expected ORs from studies with short exposure durations (and hence small shifts in the age incidence function) are too small to be detected with acceptable power.

The third fundamental problem is related to the vast diversity of tumor types to be considered. The World Health Organization differentiates about 50 types of brain tumors (Kleihues and Cavenee 2000); there are more than a dozen different histologic salivary gland tumors, and so forth. Furthermore, in recent years molecular histo pathology revealed many differences within certain types of tumors. For example, there are at least two clusters of glioblastoma multiforme, the most frequent malignant brain tumor in adults: one expresses loss of heterozygosity on chromosome 17p with mutation of the p53 tumor suppressor gene, and the other cluster is characterized by an amplification of the epidermal growth factor receptor oncogene (Schwartzbaum et al. 2006). Is it possible that all of these diverse types of tumors respond uniformly to mobile phone radiation? We cannot hope to extract sensitive types by epidemiologic investigations because of the small numbers for each distinct type.

In summary, the three most important conditions for epidemiology to arrive at a conclusion concerning a potential risk of an agent are as follows:

- It must be possible to measure (at least by a surrogate marker) the component of the agent that is related to the risk.
- For agents that promote the disease in question, duration of exposure must be a substantial fraction of the history of the disease.
- There must be good *a priori* reason to select specific types of diseases that are sufficiently homogenous to support the assumption of more or less uniform etiology.

None of these preconditions are met in the study of mobile phone use and cancer. As a consequence, even substantially increased risks might go undetected, and evidence will tend to be equivocal.

## Do Mobile Phones Cause Brain Tumors?

To assess a possible causal relationship between an agent and cancer, a pragmatic dialog approach has been delineated (Kundi 2006). According to this procedure, epidemiologic evidence must be assessed concerning four aspects: temporal relation, association, environmental, and population equivalence. If there are no valid counter arguments against the evidence for an association, this suffices for a verdict of causation. If epidemiologic evidence is insufficient, other evidence that increases or decreases confidence in a causal relationship could be included to come to a conclusion.

### Temporal relation

Assessment of temporal relation is not a trivial problem. It is impossible to define the point in time when a brain tumor started. Before a tumor can be diagnosed, which in the case of brain tumors occurs either by coincidence, if it is detected by imaging techniques applied for other reasons, or because of symptoms produced by the growing cell mass, the tumor was present for many years or even decades. For meningioma, average induction periods of about 20–40 years have been calculated in adults, based on observations of patients exposed to ionizing radiation (Umansky et al. 2008). For acoustic neuroma, slow growth, with an average volume doubling time of about 1.7 years, suggests similar induction periods (Mohyuddin et al. 2003). For glioma, case reports (Kranzinger et al. 2001) and long-term follow-up after childhood radiation therapy of tinea capitis (Sadetzki et al. 2005) also suggest induction periods of decades. Considering a temporal relationship between exposure and the diverse steps of brain tumor development, the following four phases may be differentiated: 1) exposure commenced before the first step of malignant transformation; 2) exposure started during the induction phase, which could itself last for several years; 3) onset of exposure occurred during the noninvasive growth phase; and 4) exposure started during final (autonomous) growth. In cases 1 and 2, exposure might influence malignant transformation itself and cause *de novo* occurrence of a brain tumor. In case 3, exposure might influence the fate of the deviating clone and could decrease latency or probability of spontaneous involution and therefore either increase incidence because of a shift of latency or because a tumor that would otherwise remain obscure during lifetime manifests itself clinically. In case 4, no contribution of exposure is possible. Unfortunately, little is known about the duration of these phases. Furthermore, there are likely gross differences between tumor types concerning absolute and relative length of these steps during natural history of the disease. Although slowly growing tumors such as most meningioma and schwannoma may have unchanged growth rates during prolonged periods of time, other brain tumors such as glioblastoma show an explosive final growth after possibly long periods of more stable behavior. For radiation-induced tumors, Cahan et al. (1948) proposed to allow at least 5 years for induction periods. As delineated above, for an effect during latent growth to be detected in epidemiologic studies, exposure must have been during a substantial proportion of growth phase. Therefore, for an influence both during the induction phase and on tumor growth rate, at least 5 years must be allowed for latency or duration of exposure, respectively, to fulfill the criterion of temporal relation. Because for virtually all carcinogens, repetitive or prolonged exposures are necessary to bring about an increased cancer incidence, it is necessary to consider not only time since first exposure but also duration of exposure. Number of calls and average duration of calls seem to be too difficult to remember for periods far in the past, but information about periods of regular use is more easily recalled and therefore could be the best choice for exposure determination. (In principle, it may even be validated by network provider data). Years of regular mobile or cordless phone use up to 5 years before diagnosis would possibly be the appropriate exposure meter for most slowly growing tumors. Because such evaluations have not been performed, I instead assessed exposure duration or latency of ≥ 10 years, as available, for Table 1. In these subjects, at least half of the exposure duration falls within an etiologically relevant period.

### Association

I computed a meta-analytical estimate of the risk for the different brain tumor types based on all independent studies reporting ORs for ≥ 10 years of mobile phone use. Heterogeneity was assessed by chi-square tests. A *p*-value < 0.05 was considered significant. If studies included the same population, only those without overlap were considered in the meta-analyses. Pooled effects and SEs were derived from the fixed-effect model because no significant heterogeneity was detected. Because basically all investigations used the same study design and the same end point definition, despite some deviations in exposure classification and proneness to misclassification and selection bias, no attempt was made in the meta-analysis to account for these differences. It should, however, be noted that correction for selection and misclassification bias would lead to higher meta-analytical odds ratios. There is no publication bias in this case because all studies that are planned, ongoing, or completed are known to the scientific community.

For glioma, I included three studies (Hardell et al. 2006c; Lahkola et al. 2007; Schüz et al. 2006a) reporting data on 233 exposed cases and 330 exposed controls among 2,792 glioma patients and 6,195 control subjects. I found no heterogeneity across studies, and the combined OR was 1.5 [95% confidence interval (CI), 1.2–1.8]. For acoustic neuroma, two independent pooled analyses (Hardell et al. 2006b; Schoemaker et al. 2005) gave an overall OR of 1.3 (95% CI, 0.95–1.9), based on 67 exposed cases and 311 exposed controls among 912 and 5,715 cases of acoustic neuroma and controls, respectively. Risk for meningioma from mobile phone use of ≥ 10 years was reported in two individual studies (Hardell et al. 2006b; Schüz et al. 2006a) and one pooled study (Lahkola et al. 2008), with an overall OR of 1.1 (95% CI, 0.8–1.4) evaluated from 116 exposed cases and 320 controls among a total of 2,506 meningioma patients and 6,223 control subjects. Hence, there is an increased risk for all of these end points from mobile phone use that is statistically significant for glioma. In these analyses mobile phone use was assumed to induce the neoplasia. If mobile phone use has an additional or exclusive effect on tumor growth, this analysis is not entirely appropriate because an effect on the growing tumor can only be exerted by exposure on the same side of the head where the tumor is located. Combined estimates for ipsilateral mobile phone use of ≥ 10 years give the ORs: 1.9 (95% CI, 1.4–2.4) for glioma; 1.6 (95% CI, 1.1–2.5) for acoustic neuroma; and 1.3 (95% CI, 0.9–1.9) for meningioma. Hence, there are clear indications of increased risks for all three end points.

According to the dialogue approach, association must be assessed whether or not there are valid counterarguments, and especially those based on considerations about the impact of possible biases.

### Potential biases

As apparent from Table 1, ever (regular) use of a mobile phone rarely revealed increased risks for any type of brain tumor. Except for the Finnish study of Auvinen et al. (2002), only the Swedish group of Hardell and colleagues (e.g., Hardell et al. 2002a) reported significantly elevated estimates of relative risks.

Most studies summarized in Table 1 were conducted based on the Interphone protocol (Cardis et al. 2007) that defined regular use as at least one outgoing or incoming call per week for at least 6 months, with ever-regular use starting 1 year before the reference date. Although the reference date was defined as date of diagnosis in cases and the same date of the matched control, in studies not individually matched (e.g., Hepworth et al. 2006), there are problems in defining the reference date because of the interview lag time. Because of the rapid increase in mobile phone use during and before the study period, the methods applied to compute the reference date for controls could be a source of bias. Information provided in the study reports is insufficient to decide whether adjustments were biased. In some articles (Hepworth et al. 2006; Schoemaker et al. 2005), controls were allocated into categories of interview lag time at random without consideration of age and sex of the cases within these categories, whereas in others (Lönn et al. 2004a), average lag between diagnosis and identification in a matched set was subtracted from date of control identification. The first method introduces bias if distribution of age and sex within categories of lag times differs, and the second method introduces bias if the date of identification differs between cases and controls. To my knowledge, these possible biases have not been considered previously. In the Interphone study (Cardis et al. 2007), data were collected from the end of 2000 through the beginning of 2004, with some differences between countries. During this period, mobile phone penetration rate increased from about 60% to about 90% in the European Union, according to the International Telecommunication Union. Insufficient adjustment for differences in the interview date would result in underestimation of risk.

In their studies, Hardell and colleagues disregarded mobile and cordless phone use within the last year before the reference date. Any use of a mobile or cordless phone was counted except when hands-free devices or external car antennas were used. The reference date for controls was set to the date of diagnosis of the matched case. In some reports of pooled data sets (e.g., Hardell et al. 2006c), individual matching was disregarded and controls from different studies were included. Insufficient adjustment of the reference date could have also led to bias in this case.

Hardell and colleagues defined the unexposed subjects as those who have not used a mobile or cordless phone for ≥ 1 year before diagnosis (or reference date in controls). The Interphone group disregarded cordless phones in analyses of mobile phone use (and vice versa). Cardis et al. (2007) and Takebayashi et al. (2006) have argued that cordless phone use is associated with much lower exposure to microwaves and therefore cannot be counted in exposure assessments. This view is not correct. Average power levels are not much different between cordless phones (average levels of 10 mW) and mobile phones (median average output power 6–16 mW in urban areas) (Lönn et al. 2004b). Considering the typically longer duration of daily use of cordless phones compared with mobile phones, it is not a rational procedure to exclude them from total exposure (for information on adolescent users, see Söderqvist et al. 2008). The fraction of cordless phone users among cases and controls not using mobile phones ranged from about 22% in the study of Hardell et al. (2006b) to almost 40% in the German Interphone study (Schüz et al. 2006a). If we arbitrarily assign sensitivity of exposure determination from omission of cordless phone use a value of 74% in cases and 78% in controls, assuming 100% specificity (in cases and controls) and an actual exposure prevalence of 54% [according to the data of Hardell et al. (2006b)], a true OR of 1.5 would be reduced to 1.2. Still greater reductions of the OR result if the differences in cordless phone use were actually greater.

In the Interphone studies, data acquisition concerning exposure was predominantly done by computer-assisted personal interview (CAPI). In about 95% of glioma cases and controls, exposure assessment was based on CAPI (Cardis et al. 2007). Reports from five Nordic countries (Lahkola et al. 2007) reveal that > 40% of cases were interviewed in the hospital. However, this fraction ranges from almost 100% in Finland to 3% in the United Kingdom. Data acquisition was completely different in the studies of Hardell and colleagues (Hardell et al. 2002a). They sent a questionnaire to home addresses of cases and controls, and upon return, they evaluated the questionnaires for errors, omissions, and discrepancies. If necessary, additional information was sought by telephone interviews blinded to case status.

Method of data acquisition could be important in several respects: *a*) interviews not blinded to case status may introduce a bias from the expectations of the interviewer; *b*) the interaction between interviewee and interviewer as such can lead to bias (Rosenthal effects); *c* ) answering a questionnaire at home is less demanding (especially considering the conditions after surgery) than personal interviews; *d*) at home it is possible to check telephone bills or to inspect contracts with network providers to verify data. For these reasons, the questionnaire method seems to be superior to the interview technique. However, there are also advantages of the CAPI method: data can be immediately checked for errors and discrepancies, and the interviewer can explain points that are not clear and may help in recalling inquired items. Validation studies (Berg et al. 2005; Vrijheid et al. 2006a, 2006b) within the Interphone study showed only a moderate correlation between self-reported intensity of mobile phone use and traffic data from network operators, but confirmed usage data as valid proxy for microwave exposure. For the (not very important) recent mobile phone use, self reports seem to be fairly accurate, but for earlier use, no data on reliability are available. Considering results from Christensen et al. (2005), memory performance is decreased especially in patients with high-grade glioma. Exposure assessment in these patients could be particularly biased if conducted by interviews compared with the questionnaire method. Bias often represents underreporting of mobile phone use, because it is more likely that a patient forgot using a mobile phone once years ago compared with falsely stating mobile phone use.

In the Interphone studies, overall participation was 65% for glioma cases, 78% for meningioma, and 82% for acoustic neuroma (Cardis et al. 2007). For controls, participation was 53% but there was large variation across centers, ranging from 35% to 74%. Hardell et al. (2006d), using postal questionnaires, reported participation rates of 88–91% in cases and 84–92% in controls. The participation rate in cases was computed based on eligible cases that received a questionnaire, defined as those with ascertained primary brain tumors alive at the time of identification and whose participation was not denied by their physician. If the definition of eligible cases for the Interphone studies were applied, the participation rate would amount to about 65–85% in the different studies of Hardell and colleagues. In the Interphone studies, on average, 13% (range, 2–44%) of case interviews were performed as proxy interviews. As shown by Vrijheid et al. (2006a) in an Interphone validation study, response bias due to differential selection of groups of the population with higher prevalence of mobile phone use possibly has the highest impact, even outweighing recall bias. Lönn et al. (2005) showed that non participating cases had almost the same proportion of mobile phone users (50% compared with 52% in participants), but nonparticipating controls differed markedly from participants (34% compared with 59%). Effect of this selection bias might be even greater if long-term use is considered. Consequence of selection bias can easily be determined because the biased OR is equal to the product of the true OR and the selection OR (cf. Rothman et al. 2008). Given the nonresponse analysis of Lönn et al. (2005), the selection OR is computed as 0.64 if overall participation rates of the Interphone studies are considered (72% and 53% in cases and controls, respectively). All 46 ORs in the report of Lönn et al. (2005), except one in the overall analysis of glioma and meningioma, were < 1. Assuming the selection OR is 0.75 as computed based on the participation rates for the Lönn study specifically (79% in cases and 70.5% in controls), almost all these ORs would increase above 1 and none would be significantly < 1 (as was the case for 7 of the 46 ORs). For example, the OR for > 10 years of mobile phone use for glioma, reported as 0.9, would increase to 1.2.

As has been pointed out previously (Kundi 2004; Kundi et al. 2004; Schoemaker et al. 2005), early symptoms of a developing brain tumor may have influenced behavior regarding mobile phone use. In particular, growing acoustic neuroma are frequently associated with hearing problems and tinnitus. Such symptoms may lead to a restriction of use, change of the side of the head the phone is held during calls, and even to discontinuing mobile phone use. Lönn et al. (2004a), in their study of acoustic neuroma, assessed impact of hearing loss and tinnitus 5 years before reference date and reported no differences in risk estimates for patients with and without hearing loss. Whether this is an indication that such symptoms have no impact on mobile phone use and therefore do not bias risk estimates is difficult to assess because no data were reported. As noted by Schoemaker et al. (2005), 59% of regular phone users among controls reported predominantly right-sided use, 33% left-sided use, and 8% use on both sides. The authors argued that if mobile phones cause acoustic neuromas, one might expect a higher proportion of tumors on the right than on the left side of the head among regular phone users. However, this expectation is completely unfounded. In contrast, tumor growth may cause behavioral changes, as indicated by the distribution of mobile phone use in cases, with only 49% right-sided users, 40% left-sided users, and 11% that used the phone on both sides.

Although response bias, misclassification bias, and insufficient correction of interview lag time between cases and controls will reduce risk estimates toward or even below unity, some biases could lead to a spuriously increased risk. One particular point has been raised frequently: Increased risk estimates of ipsilateral mobile phone use (Table 1) could be due to recall bias. If mobile phone use affects tumor development and growth, it is important to consider the side of the head to which the phone is held during calls. Cardis et al. (2008) reported that 97–99% of the total electromagnetic energy deposited in the brain is absorbed at the side of the head the phone is held during calls. Because of this asymmetry, an effect at the site of the growing tumor is expected only or primarily for ipsilateral use. There is no objective method to retrospectively assess side of the head the phone has been used. Asking a person about this aspect of use could result in bias. A person may be inclined to suspect mobile phone use as a causal factor and may therefore tend to report using it at the same side as the tumor has occurred. On the other hand, the reverse also may be claimed—that a person wants to dismiss the possibility that using the phone has anything to do with the disease and is therefore falsely reporting the opposite side of use. Even if a patient does not intentionally distort the answer, recent surgery may cause memory deficiencies leading to recall bias. Hepworth et al. (2006) argued that the reduced risk on the contralateral side indicates such recall bias. However, this risk reduction was due to an artifact of the method applied. Estimate of relative risk for contralateral mobile phone use was based on nonregular and ipsilateral phone users as reference. It follows that whenever the relative risk of ipsilateral phone use is > 1, the relative risk of contralateral use must be < 1 [the expected value of the OR in this case is (π_o_+π_i_)/(π_o_+ψπ_i_), where π_o_ is the proportion in the population of nonusers, π_I_ is the proportion in the population of ipsilateral users, and ψ is the OR for ipsilateral use]. All meta-analytical ORs for ipsilateral mobile phone use are > 1, and those for glioma and acoustic neuroma are statistically significant. If there was no misclassification bias in controls and perfect sensitivity, then a small recall bias in the direction of a preference for reporting mobile phone use at the side of the tumor of about 3% would reduce these enhanced ORs for long-term (≥ 10 years) use to 1. However, considering overall results for ipsilateral use, recall bias must reduce specificity in cases by 30–40% to remove the observed enhanced risk. The specificity that reduces an observed increased OR to 1 is given by [1+(ψ*− 1) π] ^−1^, where ψ* is the observed OR and π is the exposure prevalence in the population, given that sensitivity in both cases and controls and specificity in controls are 1 (Rothman et al. 2008). For example, taking the study of Hardell et al. (2005a) with an overall OR for ipsilateral mobile and cordless phone use of approximately 3.0 for acoustic neuroma and a prevalence of about 23% of ipsilateral mobile or cordless phone use, the specificity must be as low as 68% to remove the observed effect. That is, 32% of those not exposed at all or not on the side of the tumor must have falsely stated they have been exposed. In other words, more than half of mobile phone users among cases and none among controls must have given the wrong side of the head for their predominant use to remove the observed increased risk. It should also be noted that the case-only approach of Takebayashi et al. (2008) cannot solve the problem of recall bias.

As can be seen in Table 1, several Interphone groups (Christensen et al. 2005; Klaeboe et al. 2007; Lahkola et al. 2007, 2008; Lönn et al. 2005) reported ORs that were significantly < 1, implying a protective effect of mobile phone use for brain tumors. Although there is a remote possibility that mobile phone use may enhance apoptosis or activate DNA repair, such processes will hardly affect tumor development at an advanced stage; thus, this is not a valid explanation for these reduced risk estimates but rather it suggests systematic bias. Selection bias, as delineated above, could explain some of these results. However, there are likely additional biases that contributed to the overall effect. Let us consider this aspect from the perspective of the *ceteris paribus* condition (i.e., that cases and controls have essentially the same features in all relevant aspects except exposure) and in particular from the condition of population equivalence (i.e., the condition that both cases and controls stem from populations that are equivalent for all attributes that are relevant for the disease under study). Mobile phone use is not randomly distributed within the population, but usage patterns will be associated with certain attributes such as occupation, sex, socioeconomic status (SES), and age. Some of these attributes can be accounted for by matching or during analysis, but there could be an association with the disease that cannot be removed by these procedures. Exploration of prior symptoms in brain tumor cases often reveals indications of the disease process many years in the past (e.g., epileptic seizures, personality changes, a variety of cognitive and perception problems). Some of these symptoms could reduce the probability that a person chooses to use a mobile phone—or a telephone in general. Such habit changes would have the greatest impact on measures of cumulative duration and intensity of use. If aspects of the disease influence mobile phone use, the *ceteris paribus* condition is violated from the very beginning. It is evident that this also implies a violation of the condition of temporal relation, because a reversal of cause and effect may occur. These difficulties are related to the generally short duration of mobile phone use. A solution could be inclusion of case history information and formation of distinct subgroups differing in duration of symptoms related to the developing disease. Obviously, influence of symptoms on mobile phone use will predominantly reduce risk estimates, because the odds for mobile phone use in cases will be lowered.

Because of the mentioned biases that could operate in case–control studies, one may be inclined to put some weight on the only cohort study (Johansen et al. 2001; Schüz et al. 2006b) presently available. However, this investigation is severely flawed and cannot contribute to risk assessment and was therefore not included in the meta-analysis (see also Hardell et al. 2008).

### Confounding

Environmental equivalence seems to be sufficient in all investigations and confounding seems an unlikely cause of bias because there are only a few known risk factors for brain tumors that would induce bias if disregarded in the analyses. Therapeutic and, to a lesser degree, diagnostic X rays to the head region increase the risk for several types of brain tumors. In some studies this was considered a possible confounder but without effect on the risk estimates (e.g., Hardell et al. 2001), indicating that there is no correlation between such irradiation and mobile phone use. Other possible confounders include neurofibromatosis and tuberous sclerosis, family history of brain tumors, and medical treatment with growth factors, all of which are very rare conditions without reason to assume a relationship with mobile phone use. Age and sex are the most important confounders that have been considered in all studies either by matching or during analysis. SES has also been indicated as a possible confounder and was included in most analyses. However, including SES in the analysis will not remove selection bias that seems to be related to SES in some Interphone investigations (e.g., Hepworth et al. 2006).

### Assessment

Overall, arguments in favor of or against an association between mobile phone use and brain tumors are not equally strong. There is evidence for selection bias, exposure misclassification from excluding cord-less phone use, reversal of cause and effect from neglecting early symptoms of the disease, and short exposure duration. All these factors lead to reduction of the observed risk estimates. The only strong argument against an association is the possible impact of recall bias. There may be underreporting especially of early mobile phone use because of memory deficits after surgery, but most concerns have been raised for a potential distortion in reporting side of the head the phone is held during calls. Handedness correlates not very highly with side of the head the phone is used [concordances of only about 60% have been determined (Hepworth et al. 2006)], and there are no other methods at hand to validate these data. There are, however, some arguments that speak against a decisive influence of recall bias: Most participants of the Interphone and Hardell studies were enrolled during 1997–2003 at a time when mobile phone use was not widely discussed as a potential risk factor for brain tumors. As reported by Hardell et al. (2002a), among 232 brain tumor cases who expressed their views about potential causes of their disease, only two named mobile phones. Lönn (2004) asked 70 brain tumor cases and controls whether they considered mobile phones as a risk factor for brain tumors and found no difference between both groups. Even if patients consider mobile phones a factor contributing to their disease, what would they gain if they gave the wrong side of use? Most people choose to use mobile phones, but do they want to blame themselves for their disease? In several studies, mobile phone use was only one of the different risk factors investigated (e.g., Lönn et al. 2006). Bias from patients’ attribution would likely extend also to these other factors, and spuriously increased risks should have been observed in some of them. Although reporting bias of this type may explain an increased risk for long-term ipsilateral use, it is hardly an explanation for the increased overall risk of ipsilateral use revealed in several investigations (Hardell et al. 2002a, 2005a, 2006a; Hepworth et al. 2006).

When considering the discussion above, the conditions for a causal interpretation of the observed association between mobile phone use and brain tumors are not fully met. However, discussion of the potential violation of the conditions of temporal relation as well as population and environmental equivalence revealed that it must in all likelihood have reduced the observed association. Because of the important result on ipsilateral mobile phone use, a spuriously increased risk due to recall bias cannot be completely dismissed. In such a situation, evidence from other sources may increase or decrease confidence in a causal relation between mobile phone use and cancer.

### Additional evidence

Since the 1930s there has been a scientific controversy about effects of high-frequency EMF other than thermal. Absorption of electromagnetic energy is now well understood and poses no principal difficulty of integration into Maxwell’s theory of electromagnetism. The rate of absorption of electromagnetic energy in a homogenous volume of biological tissue is proportional to the temperature increase within this volume. Therefore, at high levels of EMF exposure, significant heating occurs that can be dangerous to health. Exposure standards have been issued that limit exposure to thermally safe levels. However, telecommunication and broadcasting applications of high-frequency EMF are not only radiating energy but also information. This is done by modulation of the high-frequency carrier. The modulation frequencies could be of biological significance and evoke nonthermal effects. In addition, the high-frequency signal itself could cause effects at low levels in combination with the simultaneous presence of the earth magnetic field (Chiabrera et al. 2000). In principle, interactions between EMF and matter are subject to two descriptions, classical or quantum electrodynamics. In a living cell, many important processes occur by electron transfer across membrane structures in a well-organized manner, ions cross selective channels, proteins get activated and deactivated by cascades of precisely regulated enzymes—all processes that occur on a quantum scale. Recently, by application of “omics” research (for an overview, see Vanderstraeten and Verschaeve 2008), it has been shown that cells may respond with activation of proteins and genes at nonthermal levels of exposure, a process that was also observed *in vivo* (Karinen et al. 2008). However, because of the lack of a mechanistic model, these results are uncertain as to their interpretation with respect to relevant long-term health effects. It has long been speculated (Lai and Singh 1997; Phelan et al. 1992) that free radical formation is involved in EMF-induced health effects. Although there is some evidence of formation of free radicals at nonthermal levels of EMF (Simkó et al. 2006), there are many difficulties with this approach. It would be much too simplistic to assume that free radicals are directly produced by the interaction with the EMF. Rather, these radicals are produced by the cell itself as an intermediate step of the response to sensing the field (Friedman et al. 2007).

Concerning prior epidemiologic evidence of a relationship between high-frequency EMF other than those used in mobile telecommunication and brain tumors, despite some reports of increased risk (Berg et al. 2006; Grayson 1996; Szmigielski 1996; Thomas et al. 1987), the evidence is inconclusive to date.

Results of epidemiologic studies of mobile phone use summarized above indicate an association that is of moderate strength and in the range delineated for passive smoking and lung cancer. There is no meaningful indicator of exposure dose available, but longer latencies are associated with higher risk estimates, and there are indications that risk is higher in rural areas where phones typically radiate at higher intensities (Hardell et al. 2005b). These aspects do increase confidence in a causal relationship.

In the case that epidemiology faces problems due to short exposure durations, lifetime animal bioassays gain importance in establishing a carcinogenic risk. Unfortunately, standard procedures cannot be applied for exposure to microwaves from mobile phones. In hundreds of animal carcinogenicity assays, it has been shown that even for DNA reactive agents, exposure during most of the life span of the animals at the maximum tolerated dose is necessary to significantly increase incidence. Such high exposures are impossible for high-frequency EMF because of interference with heating. Hence, it is necessary to apply levels that are much too low for an increased incidence to be expected. Some solutions to this problem have been proposed: *a* ) coexposure or prior exposure to a known carcinogen; *b*) implantation of tumor cells; and *c*) use of animal strains with a habitually increased tumor incidence. Because of the unknown mechanism of action, none of these attempts can be evaluated according to their suitability. More than 30 long-term and medium-term animal assays have been published in the past decade, most of which do not comply with basic criteria (Kundi 2003), and there is no suitable model for brain tumors. Application of ethylnitrosourea during gestation results in an increased incidence of brain tumors but increases incidence of many other tumors as well. A further problem is the much smaller size of laboratory rodents, resulting in a completely different exposure pattern at telecommunication frequencies. In contrast to humans using a mobile phone with a localized exposure at the side of the head the phone is held, animal exposure is a whole-body exposure. Although devices have been constructed that result in a predominant head irradiation (Adey et al. 2000), pattern and distribution within the brain will still be completely different from human exposures. It is not surprising then that up to now only a few animal experiments have found some indication of an increased cancer risk (e.g., Hruby et al. 2008; Repacholi et al. 1997; Shirai et al. 2007).

Interpretation of epidemiologic findings would be much easier if genotoxicity of mobile telecommunication signals could firmly be established. *In vitro* experiments have brought about diverse results that at present provide only equivocal evidence for genotoxic effects. Additionally, in the case of genotoxicity assays, procedures widely used for assessing environmental and nutritional toxicants may not be ideally suited for the study of EMF. For example, if, as suggested by Lai and Singh (2004), exposure-induced effects imply activation of the Fenton reaction, cells rich in free iron would be responsive while others would not. Hence, overall evidence without considering mechanistic hypotheses is of limited value. Nevertheless, about one quarter of published genotoxicity studies found an effect of low-level exposures (Vijayalaxmi and Obe 2004; Vijayalaxmi and Prihoda 2008).

Overall, animal and *in vitro* experiments do not reduce confidence in a causal relationship but do not provide unequivocal support either. The main problem is the lack of a coherent research strategy that unifies strengths of different disciplines to unravel the intriguing problem of low-level EMF health effects from a biophysical perspective.

## Conclusions

Epidemiologic evidence compiled in the past 10 years starts to indicate an increased risk, in particular for brain tumors (glioma, meningioma, acoustic neuroma), from mobile phone use. Considering biases that may have been operating in most studies, the risk estimates are rather too low, although recall bias could have increased risk estimates. The net result, when considering the different errors and their impact, is still rather an elevated risk. The magnitude of the brain tumor risk is moderate, but it has to be borne in mind that estimates are still from short durations of exposure. From the perspective of public health, an increase of brain tumor incidence of ≥ 50% poses substantial problems for neurosurgical care, but the individual perspective is less dramatic: in industrial countries, the lifetime brain tumor risk is 4–8 per 1,000. If mobile phone use should increase these figures to 6–12 per 1,000, the individual risk is still low.

At present, evidence for a causal relationship between mobile phone use and brain tumors relies predominantly on epidemiology, in particular on the large studies of Hardell and colleagues, but there are no valid counter arguments and no strong evidence decreasing confidence in a causal relationship. Weak evidence in favor of a causal relationship is provided by some animal and *in vitro* studies, but overall, genotoxicity assays, both *in vivo* and *in vitro*, are inconclusive to date.

## Figures and Tables

**Table 1:** Overview of results [odds ratios or standardized incidence ratios (95% CIs)] from epidemiologic studies of mobile phone use and brain tumors.

Study	Type	Cohort size (no. of cases and controls)	Average duration of MP use (years)	Overall results	Results for longer duration of MP use	Result for ipsilateral MP use
Dreyer et al. 1999	Cohort	133,423 Hand-held MP	~ 2	2 Brain tumor deaths	—	—
		152,138 Portable bag		4 Brain tumor deaths		
Hardell et al. 1999	Case–control	209 Brain tumor cases	~ 6	0.98 (0.69–1.41)	> 10 years, 1.20 (0.56–2.59)	2.42 (0.97–6.05)
Hardell et al. 2000						
Hardell et al. 2001	425 Controls					2.62 (1.02–6.71) (multiv)
Muscat et al. 2000	Case–control	469 Malignant brain tumor cases	~ 3	0.85 (0.6–1.2)	≥ 4 years, 0.7 (0.3–1.4)	2.01 (0.92–5.89)
		422 Controls				
Inskip et al. 2001	Case–control	489 Glioma	~ 3	1.0 (0.7–1.4)	≥ 5 years, 0.6 (0.3–1.4)	0.86 (0.65–1.35) (overall)
		197 Meningioma		0.8 (0.5–1.2)	0.9 (0.3–2.7)	
		96 Acoustic neuroma		0.8 (0.5–1.4)	1.9 (0.6–5.9)	
		799 Controls				
Johansen et al. 2001	Retrospective cohort	420,095 Subscribers	2001, ~ 3	1.0 (0.8–1.1)	≥ 3 years, 1.2 (0.6–2.3)	—
Schüz et al. 2006b			2006, ~ 8	1.0 (0.9–1.1)	≥ 10 years, 0.66 (0.44–0.95)	
Auvinen et al. 2002	Case–control	398 Brain tumor cases	Analog, ~ 2.5	1.6 (1.1–2.3)	> 2 years, 1.6 (0.9–2.8)	—
		1,986 Controls	Digital, ~ 1	0.9 (0.5–1.5)	0.6 (0.1–4.5)	
Muscat et al. 2002	Case–control	90 Acoustic neuroma	~ 3	0.68 (0.34–1.38)	≥ 3 years, 1.7 (0.5–5.1)	0.55 (0.50–1.05)
		86 Controls				
Hardell et al. 2002a	Case–control	1,303 Brain tumor cases	Analog, ~ 7	1.3 (1.02–1.6)	> 10 years, 1.8 (1.1–2.9)	1.8 (1.3–2.5)
			Digital, ~ 4,	1.0 (0.8–1.2)	—	1.3 (0.99–1.8)
			Cordless, ~ 6	1.0 (0.8–1.2)	2.0 (0.5–8.0)	1.3 (1.01–1.8)
		611 Meningioma	Analog, ~ 7	1.1 (0.7–1.5)	> 10 years, 1.0 (0.1–16.0)	—
			Digital, ~ 4	0.8 (0.6–1.03)	—	
			Cordless, ~ 6	0.9 (0.6–1.1)	—	
		159 Acoustic neuroma	Analog, ~ 7	3.5 (1.8–6.8)	> 10 years, 3.5 (0.7–16.8)	—
			Digital, ~ 4	1.2 (0.7–2.2)	—	
		1,303 Controls	Cordless, ~ 6	1.0 (0.6–1.7)	2.0 (0.2–22.0)	
Hardell et al. 2002b^a^	Case–control	588 Malignant brain tumor cases	Analog, ~ 7	1.13 (0.82–1.57)	> 6 years, 1.17 (0.75–1.81)	1.80 (0.96–3.38) > 6 years
			Digital, ~ 4	1.13 (0.86–1.48)	1.71 (0.67–4.34)	2.29 (0.59–8.93)
		581 Controls	Cordless, ~ 5	1.13 (0.85–1.50)	1.56 (0.92–2.63)	1.16 (0.55–2.46)
Christensen et al. 2004	Case–control	106 Acoustic neuroma	~ 4	0.90 (0.51–1.57)	≥ 10 years, 0.22 (0.04–1.11)	0.68 (0.58–0.90)
		212 Controls				
Lönn et al. 2004a	Case–control	148 Acoustic neuroma	~ 5	1.0 (0.6–1.5)	≥ 10 years, 1.9 (0.9–4.1)	3.9 (1.6–9.5) ≥ 10 years
		604 Controls				
Christensen et al. 2005	Case–control	175 Meningioma	~ 5	0.83 (0.54–1.28)	≥ 10 years, 1.02 (0.32–3.24)	
		81 Glioma I-II		1.08 (0.58–2.00)	1.64 (0.44–6.12)	
		171 Glioma III-IV		0.58 (0.37–0.90)	0.48 (0.19–1.26)	
		822 Controls				
Hardell et al. 2006a	Case–control	317 Malignant brain tumor cases	Analog, ~ 10	2.6 (1.5–4.3)	> 10 years, 3.5 (2.0–6.4)	3.1 (1.6–6.2)
			Digital, ~ 6	1.9 (1.3–2.7)	3.6 (1.7–7.5)	2.6 (1.6–4.1)
		692 Controls	Cordless, ~ 6	2.1 (1.4–3.0)	2.9 (1.6–5.2)	2.9 (1.8–4.7)
Hardell et al. 2005a	Case–control	305 Meningioma	Analog, ~ 9	1.7 (0.97–3.0)	> 10 years, 2.1 (1.1–4.3)	1.6 (0.7–3.9)
			Digital, ~ 5	1.3 (0.9–1.9)	1.5 (0.6–3.9)	1.5 (0.9–2.5)
			Cordless, ~ 5	1.3 (0.9–1.9)	1.9 (0.97–3.6)	1.6 (0.97–2.6)
		84 Acoustic neuroma	Analog, ~ 9	4.2 (1.8–10)	> 10 years, 2.6 (0.9–8)	5.1 (1.9–14)
			Digital, ~ 5	2.0 (1.05–2.8)	0.8 (0.1–6.7)	2.9 (1.4–6.1)
		692 Controls	Cordless, ~ 5	1.5 (0.8–2.9)	0.3 (0.03–2.2)	2.4 (1.1–5.1)
Lönn et al. 2005	Case–control	371 Glioma	Analog, ~ 9	0.8 (0.5–1.2)	≥ 10 years, 0.8 (0.5–1.5)	1.6 (0.8–3.4) ≥ 10 years
			Digital, ~ 3	0.8 (0.6–1.0)	≥ 5 years, 0.8 (0.6–1.2)	
		273 Meningioma	Analog, ~ 9	0.7 (0.4–1.3)	≥ 10 years, 0.9 (0.5–2.0)	1.3 (0.5–3.9) ≥ 10 years
		674 Controls	Digital, ~ 3	0.6 (0.5–0.9)	≥ 5 years, 0.8 (0.5–1.2)	
Schoemaker et al. 2005^b^	Case–control	678 Acoustic neuroma	Analog, ~ 8	0.9 (0.7–1.2)	≥ 10 years, 1.1 (0.7–1.7)	1.8 (1.1–3.1) ≥ 10 years
		3,553 Controls	Digital, ~ 4	0.9 (0.7–1.1)	0.7 (0.2–3.5)	
Hepworth et al. 2006	Case–control	966 Glioma	~ 5	0.94 (0.78–1.13)	≥ 10 years, 1.14 (0.74–1.73)	1.24 (1.02–1.52)
		1,716 Controls				
Schüz et al. 2006a	Case–control	366 Glioma	~ 4	0.98 (0.74–1.29)	≥ 10 years, 2.20 (0.94–5.11)	
		381 Meningioma		0.84 (0.62–1.13)	1.09 (0.35–3.37)	
		1,494 Controls				
Klaeboe et al. 2007	Case–control	289 Glioma	~ 4	0.6 (0.4–0.9)	≥ 6 years, 0.8 (0.5–1.2)	1.2 (0.7–1.2) ≥ 6 years
		207 Meningioma		0.8 (0.5–1.1)	1.0 (0.6–1.8)	1.4 (0.7–2.9)
		45 Acoustic neuroma		0.5 (0.2–1.0)	0.5 (0.2–1.5)	0.7 (0.2–2.5)
		358 Controls				
Takebayashi et al. 2006	Case–control	101 Acoustic neuroma	~ 4	0.73 (0.43–1.23)	≥ 8 years, 0.79 (0.24–2.65)	0.90 (0.50–1.62)
		339 Controls				
Lahkola et al. 2007 c	Case–control	1,521 Glioma	~ 6	0.78 (0.68–0.91)	≥ 10 years, 0.95 (0.74–1.23)	1.39 (1.01–1.92) ≥ 10 years
		3,301 Controls				
Schlehofer et al. 2007	Case–control	97 Acoustic neuroma	~ 4	0.67 (0.38–1.19)		
		194 Controls				
Hours et al. 2007	Case–control	96 Glioma		1.15 (0.65–2.05)	> 4 years, 1.96 (0.74–5.20)	
		96 Controls				
		109 Acoustic neuroma		0.92 (0.53–1.59)	> 4 years, 0.66 (0.28–1.57)	
		214 Controls				
Takebayashi et al. 2008	Case–control	88 Glioma	~ 4	1.22 (0.63–2.37)	> 6.5 years, 0.60 (0.20–1.78)	1.24 (0.67–2.29)
		132 Meningioma		0.70 (0.42–1.16)	> 5.2 years, 1.05 (0.52–2.11)	1.14 (0.65–2.01)
		392 Controls				
Lahkola et al. 2008 d	Case–control	1,209 Meningioma	5.5	0.76 (0.65–0.89)	≥ 10 years, 0.85 (0.57–1.26)	0.99 (0.57–1.73) ≥ 10 years
		3,299 Controls				

Abbreviations: CI, confidence interval; MP, mobile phone; multiv, multivariate.

a Data are a subset from Hardell et al. (2002a).

b Includes data from Christensen et al. (2004) and Lönn et al. (2004a).

c Includes data from Lönn et al. (2005), Christensen et al. (2005), Hepworth et al. (2006), and Klaeboe et al. (2007).

d Includes data from Lönn et al. (2005), Christensen et al. (2005), and Klaeboe et al. (2007).
